# Expression of the Mismatch Repair Gene hMLH1 Is Enhanced in Non-Small Cell Lung Cancer with EGFR Mutations

**DOI:** 10.1371/journal.pone.0078500

**Published:** 2013-10-24

**Authors:** Mei Li, Qiuping Zhang, Lina Liu, Weipeng Lu, Hong Wei, Rachel W. Li, Shen Lu

**Affiliations:** 1 Central Laboratory, The Second Hospital of Dalian Medical University, Dalian, PR China; 2 Department of Pathology, The First Hospital of Dalian Medical University, Dalian, PR China; 3 Department of Internal Medicine, The First Hospital of Dalian Medical University, Dalian, PR China; 4 The Medical School, The Australian Medical University, Canberra, Australia; University General Hospital of Heraklion and Laboratory of Tumor Cell Biology, School of Medicine, University of Crete, Greece

## Abstract

Mismatch repair (MMR) plays a pivotal role in keeping the genome stable. MMR dysfunction can lead to carcinogenesis by gene mutation accumulation. HMSH2 and hMLH1 are two key components of MMR. High or low expression of them often mark the status of MMR function. Mutations (EGFR, KRAS, etc) are common in non-small cell lung cancer (NSCLC). However, it is not clear what role MMR plays in NSCLC gene mutations. The expression of MMR proteins hMSH2 and hMLH1, and the proliferation markers PCNA and Ki67 were measured by immunohistochemistry in 181 NSCLCs. EGFR and KRAS mutations were identified by high resolution melting analysis. Stronger hMLH1 expression correlated to a higher frequency of EGFR mutations in exon 19 and 21 (p<0.0005). Overexpression of hMLH1 and the adenocarcinoma subtype were both independent factors that related to EGFR mutations in NSCLCs (p=0.013 and p<0.0005). The expression of hMLH1, hMSH2 and PCNA increased, while Ki67 expression significantly decreased (p=0.030) in NSCLCs with EGFR mutations. Overexpression of hMLH1 could be a new molecular marker to predict the response to EGFR-TKIs in NSCLCs. Furthermore, EGFR mutations might be an early event of NSCLC that occur before MMR dysfunction.

## Introduction

 Lung cancer is the most frequent and deadly malignant tumor worldwide, with non-small cell lung cancer (NSCLC) being the predominant form. Carcinogenesis of NSCLC is a multistep process involving alterations of multiple genes including oncogene activation and tumor suppressor gene inactivation [1]. Recent development of new agents with specific molecular targets, especially epidermal growth factor receptor tyrosine kinase inhibitors (EGFR-TKIs), has enhanced scientific interest in particular gene mutations and challenged some of the established paradigms in the therapeutic intervention of NSCLC [2]. The EGFR signal transduction pathway is one of the main pathways that participate in the mediation and regulation of cell proliferation [3]. The cells proliferate rampantly with malignant transformation [4,5]. Approximately 30-50% of NSCLCs have mutations in key genes, such as EGFR, KRAS, BRAF, PI3K and AKT. The two most commonly mutated oncogenes are EGFR and KRAS [6,7]. These gene mutations are often related to the NSCLC patient response to molecular targeted drugs. For example, tumors with EGFR mutations in exon 19 or 21 are often sensitive to EGFR TKIs. In contrast, patients with mutant KRAS tumors fail to benefit from adjuvant chemotherapy and do not respond to EGFR inhibitors [8–10]. Interestingly, half of the NSCLCs with the mutation in EGFR exon 19 or exon 21 produce secondary mutations in EGFR exon 20 and become resistant to TKIs after treatment for one year [11,12]. This indicates that the key genes of the EGFR pathway are unstable in NSCLC. Not only is there a higher mutation frequency in NSCLC, but also some genes like EGFR can easily produce secondary mutations. However, it is not clear if gene mutations in NSCLC are related to abnormalities in the DNA repair mechanism.

 Mismatch repair (MMR) is an important type of DNA repair, playing a pivotal role in maintaining genome stability [13]. The hMSH2 and hMLH1 genes, which are the key components of the MMR system, recognize and excise single-base mismatches and insertion/deletion loops that occur during DNA replication or DNA damage [14]. MMR dysfunction often leads to genomic instability, including microsatellite instability (MSI) and the accumulation of gene mutations, which are thought to be associated with carcinogenesis of various malignant tumors [15,16]. The dysregulation of hMLH1 or hMSH2 expression, usually from a loss of heterozygosity (LOH) at the DNA MMR loci, by mutation or promoter methylation, is the main reason for MMR dysfunction [17,18]. The loss of hMLH1 or hMSH2 expression is associated with a hypermutation phenotype, including KRAS, BRAF, APC, P53, and TGF-β mutations in colorectal cancer [19–22]. It is not clear, however, that MMR affects gene mutations in NSCLC. In order to study the correlation between MMR and NSCLC mutations, we detected EGFR and KRAS mutations and measured hMLH1, hMSH2, PCNA and Ki67 expression in NSCLC tumors.

## Materials and Methods

### 2.1: Ethics Statement

 The study was approved by the Ethics Committee of the Second Hospital of Dalian Medical University. All specimens in the research were from tissue surgically removed without affecting the diagnosis and treatment. They were collected with the written informed consent of the patients or families before surgery. The data were analyzed anonymously. All procedures were in accordance with the Declaration of Helsinki.

### 2.2: Patients and tumor specimens

 A total of 181 tumor specimens were collected from NSCLC patients who underwent surgical procedures at the affiliated hospitals of Dalian Medical University from 2007 to 2009. Of these, there were 112 adenocarcinomas, 58 squamous cell carcinomas, 4 adeno-squamous cell carcinomas, 5 large cell carcinomas and 2 sarcomatoid carcinomas. Two certified pathologists independently diagnosed and classified all the patients according to the WHO classification (2004). Of the 181 patients studied, 109 were men and 72 were women with a mean ± SD age of 62.0 ± 9.3 years (36-80 years). None of the patients received radio- or chemotherapy before their operations. The patients' information and histopathological features of the tumors in this cohort are presented in Table 1. Each tumor specimen was divided into two parts. One portion was quickly frozen for sectioning and DNA extraction, the other portion was formalin-fixed and paraffin-embedded for immunohistochemistry. 

**Table 1:** Correlation of clinicopathological parameters, immunohistochemical expression and gene mutations in NSCLC.

**Variables**	**No.**	**hMSH positive (%)**	**hMLH1 positive (%)**		**PCNA positive (%)**	**ki67 positive (%)**	**EGFR exon 19 Mutation (%)**		**EGFR exon 21 Mutation (%)**		**KRAS exon 2 Mutation (%)**	
Age												
≤60	79	59.5	68.4		88.6	57.0	12.7		25.3		5.1	
>60	102	54.9	73.5		88.2	65.7	13.7		21.6		5.9	
Gender												
Female	72	54.2	79.2		90.3	55.6	20.8	a	37.5	c	0.0	b
Male	109	58.7	66.1		87.2	66.1	8.3		13.8		9.2	
Pathology												
Adc	112	56.3	77.7	a*	90.2	56.3	21.4	c	32.1	c	5.4	
SCC	58	56.9	62.1		87.9	70.7	0.0		5.2		6.9	
Smoking												
Non-smoking	115	56.5	78.3	b*	88.7	60.0	17.4	a	30.4	c	3.5	
Smoking	66	57.6	59.1		87.9	65.2	6.1		10.6		9.1	
Tumor site												
Left lung	85	55.3	76.5		88.2	57.6	17.6		18.8		8.2	
Right lung	96	58.3	66.7		88.5	65.6	9.4		27.1		3.1	
LN metastasis												
No	86	61.6	72.1		88.4	65.1	12.8		19.8		8.1	
Yes	95	52.6	70.5		88.4	58.9	13.7		26.3		3.2	
Stage												
I & II	111	61.3	74.8		91.0	61.3	16.2		23.4		5.4	
III & IV	70	50.0	65.7		84.3	62.9	8.6		22.9		5.7	

Adc: adenocarcinoma; SCC: squamous cell carcinoma; LN: lymph node.

^a^ p<0.05, ^b^p<0.01, ^c^p<0.0005 (Pearson chi-square test).

^*^ When smoking history was controlled, hMLH1 expression is not significantly different between Adc and SCC, p=0.267; when pathological classification was controlled, it is different between non-smokers than smokers, p= 0.009 (CMH test).

### 2.3: Immunohistochemical analysis

 Monoclonal antibodies against human hMSH2 (1:250, clone FE11, Invitrogen, Life technologies, USA), hMLH1 (1:50, clone 14, Invitrogen, Life technologies, USA), PCNA (1:400, clone PC10, Thermo scientific, USA) and Ki67 (1:100, clone K-2, Invitrogen, Life technologies, USA) were used as primary antibodies. Biotin-streptavidin-peroxidase staining with 3, 3’-diaminobenzidine-tetrahydrochloride (DAB) detection were used. Immunohistochemistry was performed as previously described [23]. Tumor cells with staining in the nuclei were considered positive. Each slide was graded blindly according to the percentage of positive tumor cells (0-5%, 5-10%, 10-25%, 25-50%, 50-100%) and the intensity of staining (none, weak, moderate and strong) by two independent pathologists [24–28]. In most slides the expression intensity was related to the expression frequency. Immunoreactivity of hMSH2, hMLH1, PCNA and Ki67 was evaluated as negative (-), positive tumor cells less than 25%; positive (+), 25-50% positive tumor cells; and strong positive (++), ≥ 50% positive tumor cells. 

### 2.4: DNA extraction and gene mutation detection

 Tumor enriched areas were selected and cut from the stained frozen sections marked by two pathologists. Genomic DNA was extracted from these areas and purified according to the manufacturer’s protocol (Tiangen, Beijing, China) [29,30]. KRAS exon 2 and EGFR exon 19 and 21 of each specimen were amplified in triplicate in a 10 µL reaction volume with a 15 µL mineral oil overlay in each well of a 96-well PCR plate on a Mastercycler (Eppendorf, German). The primers were 5'-AGGCCTGCTGAAAATGACT-3' and 5'-AATGGTCCTGCACCAGTAA-3' (KRAS exon 2); 5'-TGGATCCCAGAAGGTGAGAA -3' and 5'-AGCAGAAACTCACATCGAGGA -3' (EGFR exon 19); 5'-CGCAGCATGTCAAGATCA -3' and 5'-CCTCCTTACTTTGCCTCC -3' (EGFR exon 21). The reaction conditions were as previously reported [29,30]. The mutations were detected with high resolution melting analysis on a LightScanner^®^ 96 (Biofire Diagnostics, USA). The melting curves were acquired from 60 °C to 95 °C, and analyzed using LightScanner software (version 2.0) according to the manufacturer’s instructions [29,30]. 

### 2.5: Statistical analysis

 The Pearson chi-square test and Fisher’s exact test were used to compare the difference of protein expression between clinicopathological parameters. Spearman’s correlation analysis was used to test the correlation between protein expression. The Cochran’s and Mantel-Haenszel (CMH) test was used to compare the difference of hMLH1 expression between smoking status and between the tumor classifications, with the other variable controlled. Logistic regression was used to analyze the factors related to EGFR mutations. All of the analyses were performed with SPSS 13.0 at the significance level of p<0.05.

## Results

### 3.1: Expression of hMSH2, hMLH1, PCNA and Ki67 in NSCLCs and clinicopathological parameters

**Figure 1:**
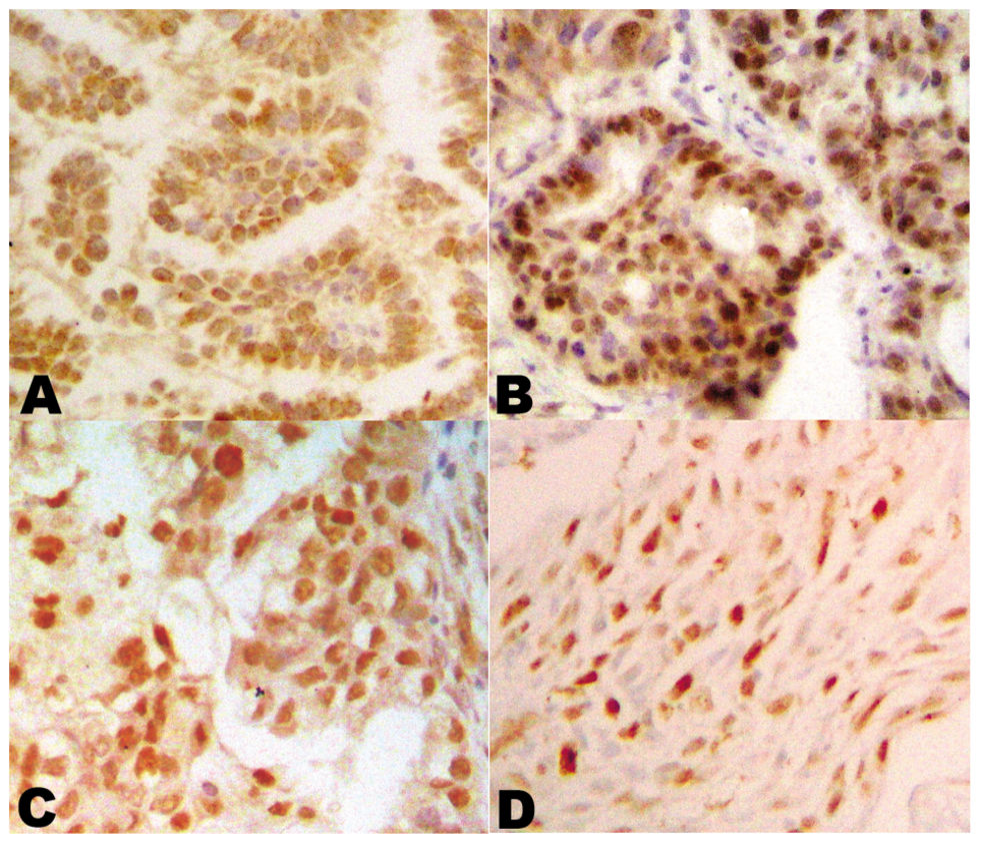
Protein expression of hMLH1, hMSH2, PCNA and Ki67 in NSCLCs. Immunohistochemical profiling of hMLH1 protein positive expression (A), hMSH2 protein positive expression (B), PCNA protein positive expression (C) and Ki67 protein positive expression (D). (×200).

 All of the proteins, hMSH2, hMLH1, PCNA and Ki67, were expressed in the nuclei of tumor cells (Figure 1). HMSH2, hMLH1, PCNA and Ki67 were expressed in 59.6%, 71.3%, 88.4% and 61.9% of the tumors respectively. Protein expression between clinicopathological groups is presented in Table 1. There was a higher frequency of hMLH1 expression in non-smokers compared to smokers (p=0.006). Similarly, a higher frequency of expression was observed in adenocarcinoma compared to squamous cell carcinoma (p=0.031). But there was no significant difference of hMLH1 expression between adenocarcinoma and squamous cell carcinoma when the factor of smoking history was controlled (p=0.267), while a significant difference was found when the pathological classification was controlled (p=0.009). This suggests that hMLH1 expression is principally affected by smoking history, not pathological classification.

### 3.2: Correlation among hMSH2, hMLH1, PCNA and Ki67 expression

 There were 81 cases with hMSH2 and hMLH1 co-expression, 22 cases with only hMSH2 expression, 48 cases with only hMLH1 expression and 30 cases without a positive expression of either hMSH2 or hMLH1. The hMSH2 expression was significantly correlated to the hMLH1 expression (p=0.038; r=0.155). The expression of hMLH1 was stronger in the cases with PCNA expression (p=0.005), but not in those with Ki67 expression (p=0.495). There was a trend of hMLH1 expression increasing with PCNA expression (p=0.056). Expression of hMSH2 was not correlated to the expression of either PCNA or Ki67 (p=0.802; p=0.099) (Table 2).

**Table 2:** Correlation of hMLH1, hMSH2, and PCNA and Ki67 expressions.

		hMSH2				PCNA				Ki67		
		-	+	++		-	+	++		-	+	++
hMLH1	-	31	4	18	a	12	21	20	b	9	28	6
	+	21	6	35		6	40	16		26	31	5
	++	26	4	36		3	34	29		24	30	12
hMSH2	-					9	39	30		34	36	8
	+					3	7	4		7	5	2
	++					9	49	31		28	48	13

^a^ p<0.05 (Spearman’s correlation analysis), Spearman’s rank correlation coefficient (r) is 0.155.

^b^ p=0.056 (Spearman’s correlation analysis), p=0.005 (Pearson chi-square test).

### 3.3: KRAS and EGFR mutations in NSCLCs

**Figure 2:**
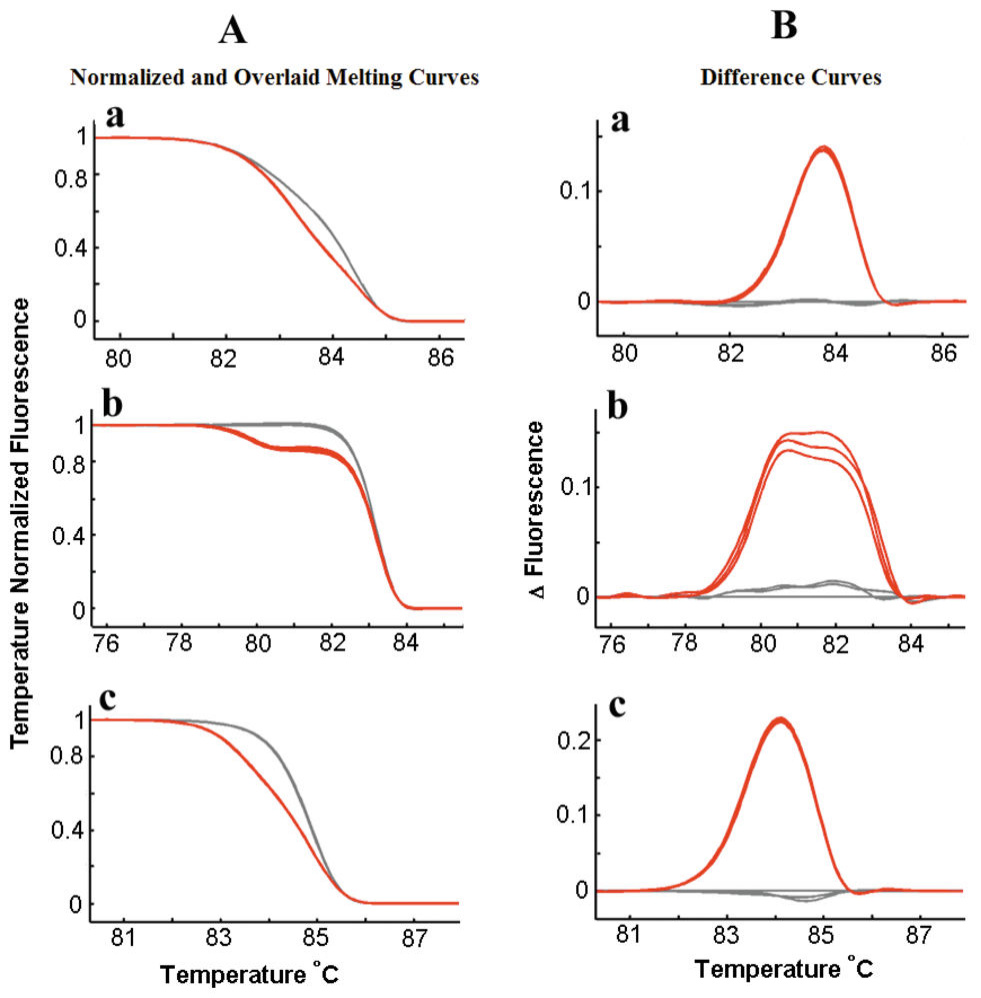
EGFR, KRAS mutation detection with high resolution melting analysis. Different melting curves showing mutation type (red line) relative to wild type (grey line) of KRAS exon 2 (a), EGFR exon 19 (b) and EGFR exon 21 (c). Every sample was analyzed in triplicate. The data was plotted directly (A) or the wild type was chosen as a horizontal base line (B).

 Out of the 181 patients with NSCLCs, there were 10 cases (5.5%) with a KRAS mutation and 66 cases (36.5%) with an EGFR mutation (24 cases in exon 19 and 42 cases in exon 21) (Figure 2). KRAS mutations were more frequent in men than in women (p=0.008). There was no significant correlation of KRAS mutations with other clinicopathological features (Table 1). The frequency of EGFR mutations, either in exon 19 or exon 21, was higher in women than in men (p=0.015; p<0.0005), in adenocarcinoma than in squamous cell carcinoma (p<0.0005; p<0.0005), and in the non-smokers than in smokers (p=0.031; p=0.002). There was no significant correlation of EGFR mutations to patient age, lymph node metastasis, tumor site or clinical stage (Table 1). 

### 3.4: Correlation of KRAS and EGFR mutations with the expression of hMSH2, hMLH1, PCNA and Ki67 in NSCLC

 There was no significant difference in the frequency of Ki67 or PCNA expression between NSCLCs with and without EGFR mutation in exon 19 or exon 21 (p>0.05, Table 3). But Ki67 expression was less frequent in NSCLCs with EGFR mutations (both in exon 19 and 21) than in those without the mutations (51.5% to 67.8%, p=0.030), but PCNA was not (85.2% to 93.9%, p=0.078). 

**Table 3:** Correlation of hMSH2, hMLH1, PCNA and Ki67 expression with KRAS and EGFR mutations.

		***n***	**hMSH2 (%)**	**hMLH1 (%)**		**PCNA (%)**	**Ki67 (%)**
KRAS	M	10	60.0	80.0		80.0	40.0
	W	171	56.7	70.8		88.9	63.2
EGFR exon 19	M	24	70.8	91.7	a	95.8	45.8
	W	157	54.8	68.2		87.3	64.3
EGFR exon 21	M	42	52.4	88.1	b	92.9	54.8
	W	139	58.3	66.2		87.1	64.0

M: mutation, W: wild type.

^a^ p<0.05, ^b^ p<0.01 (Pearson chi-square test or Fisher’s exact test).

 The frequency of hMLH1 expression was higher in NSCLCs with an EGFR exon 19 mutation than in those without the mutation (91.7% to 68.2%, p=0.018) and in NSCLCs with an EGFR exon 21 mutation than in those without the mutation (88.1% to 66.2%, p=0.006). As hMLH1 expression increases (from -, + to ++), the frequency of EGFR mutations (exon 19 and 21) were 13.2%, 38.7% and 53.0% respectively (p<0.0005). Similar correlations were not found with hMSH2 expression (Table 3). The adenocarcinoma subtype and hMLH1 overexpression were two independent factors that relate to EGFR mutations (p<0.0005 and p=0.013), but gender and smoking history do not (p=0.070 and p=0.538).

## Discussion

 Molecular targeting of drugs is beginning to play a more important role in tumor treatment. To improve clinical results for patients with NSCLC, targeted therapies are increasingly being used with encouraging outcomes, particularly in patients with specific molecular features [31]. EGFR and KRAS mutations are two well-known markers that indicate the sensitivity and resistance to EGFR-TKIs of NSCLC patients. The type of mutation varies between ethnic groups. For example, the frequency of EGFR mutations is higher in East Asians with NSCLC than in Caucasians. In contrast to EGFR mutations, KRAS mutations are found in 20-30% of Caucasians, while in less than 10% of East Asians [29,30,32–36]. However, many NSCLC patients do not have EGFR or KRAS mutations. So their response to EGFR-TKIs cannot currently be predicted. Therefore, it is necessary to find new molecular markers to predict the response of NSCLC patients to these drugs. 

 To the best of our knowledge, we report here for the first time that hMLH1 expression is related to EGFR mutations in both exon 19 and exon 21, but hMSH2 expression is not. Generally, women and non-smoking patients with adenocarcinoma have a relatively high probability of EGFR mutations. But lung adenocarcinoma is common in women and non-smokers, and most women in East Asia are non-smokers. Therefore, clinicopathological characteristics do not predict EGFR mutations very well. We found hMLH1 expression and adenocarcinoma were independent factors related to EGFR mutations. Moreover, the stronger the hMLH1 expression, the higher EGFR mutation frequency. Gender and smoking history were not independently correlated to EGFR mutation frequency. It would be interesting to study the value of hMLH1 overexpression as a marker to predict the response of NSCLC patients to EGFR-TKIs.

 In previous studies, Xinarianos et al. reported that lower hMLH1 expression was more frequent in heavy smokers [27]. HMSH2 and hMLH1 expression were also different in adenocarcinomas compared to squamous cell carcinomas [27]. Vageli et al. evaluated the mRNA level of hMSH2 and hMLH1 in 29 primary NSCLCs and found the frequency of hMLH1 mRNA expression was higher in non-smokers than in smokers. This study also found that there were differences in the expression pattern of hMLH1 and hMSH2 between adenocarcinoma and squamous cell carcinoma [37,38]. Wang et al. found that there was more hMLH1 and hMSH2 expression in NSCLC samples from women than in those from men [39]. We found hMLH1 expression was higher in patients without smoking history. But it was not different between adenocarcinoma and squamous cell carcinoma and between genders, when we adjusted with the factor of smoking history. It suggests that smoking could be a major factor that affects hMLH1 expression. Saletta et al. and Vogelsang et al. independently found that exposure to tobacco smoke inactivates MMR function by inducing chromosomal instability and polymorphisms of the hMLH1 gene [40,41]. 

 Both PCNA and Ki67 can be used to indicate the status of cell proliferation. PCNA is stimulated in the process of MMR as a necessary component [21], while Ki67 not. In this study, we found cases with EGFR mutations have a higher frequency of both hMLH1 and PCNA expression, but a trend toward lower Ki67 expression. This suggests that an EGFR mutation might stimulate and initiate the process of DNA repair by increasing hMLH1 and PCNA expression, and then prolong the cell cycle. Therefore, EGFR mutations in NSCLCs would activate the MMR function, instead of being the result of genomic instability caused by MMR dysfunction. EGFR mutations might be an early event in the carcinogenesis of NSCLC before MMR dysfunction. 

In addition, Kouso et al. demonstrated the independence of hMSH2 and hMLH1 expression with different roles in NSCLC [28]. Besides a role in the process of MMR as a key component, the hMLH1 protein also interacts with other DNA repair and apoptosis signaling molecules such as PCNA, BRCA1, P53 and ATM [42–45]. Therefore, hMLH1 might be also regulated by other factors. An et al. and Shih et al. reported that specific polymorphisms of hMLH1 are related to the susceptibility and prognosis of lung cancer and occurred more often in lung squamous cell carcinoma than in adenocarcinoma [46,47]. All of these factors could lead to imbalance of hMSH2 and hMLH1 expression. Moreover, hMSH2 and hMLH1 expression can vary not only between different histological origins, but also between different ethnic groups [32–36].

In summary, EGFR mutations in exon 19 and 21 correlate with MMR dysfunction in NSCLC. Overexpression of hMLH1 could be a new marker for patient sensitivity to EGFR-TKIs. In the past, MMR dysfunction has been assumed to cause EGFR mutations. However, EGFR mutations could also increase hMLH1 overexpression as a compensatory mechanism. A cause-effect relationship has not been established either way. Further studies would be required to provide further insight into which event occurs first. In other case, the possibility of using hMLH1 as an indicator of TKI responses may prove useful.
